# NCOA1 promotes angiogenesis in breast tumors by simultaneously enhancing both HIF1α- and AP-1-mediated VEGFa transcription

**DOI:** 10.18632/oncotarget.4341

**Published:** 2015-06-20

**Authors:** Li Qin, Yan Xu, Yixiang Xu, Gang Ma, Lan Liao, Yelin Wu, Yi Li, Xian Wang, Xiaosong Wang, Jun Jiang, Jin Wang, Jianming Xu

**Affiliations:** ^1^ Department of Molecular and Cellular Biology, Baylor College of Medicine, Houston, TX, USA; ^2^ Department of Breast and Thyroid Surgery, Daping Hospital, Third Military Medical University, Chongqing, China; ^3^ Breast Disease Center, Southwest Hospital, Third Military Medical University, Chongqing, China; ^4^ Institute of Biosciences and Technology, Texas A&M University Health Science Center, Houston, TX, USA; ^5^ Shanghai Key Laboratory of Regulatory Biology, Institute of Biomedical Sciences, School of Life Sciences, East China Normal University, Shanghai, China; ^6^ Lester and Sue Smith Breast Center, Baylor College of Medicine, Houston, TX, USA; ^7^ Department of Pharmacology, Baylor College of Medicine, Houston, TX, USA; ^8^ Institute for Cancer Medicine and College of Basic Medical Sciences, Sichuan Medical University, Luzhou, Sichuan, China

**Keywords:** NCOA1, VEGFa, transcriptional regulation, breast cancer

## Abstract

Nuclear receptor coactivator 1 (NCOA1) is overexpressed in a subset of breast cancer and its increased expression positively correlates with disease recurrence and metastasis. Although NCOA1 is known to promote breast cancer metastasis through working with multiple transcription factors to upregulate the expression of Twist1, ITGA5, CSF-1, SDF1 and CXCR4, the role of NCOA1 in breast tumor angiogenesis has not been investigated. In this study, we found that the microvascular density (MVD) was significantly decreased and increased in Ncoa1-knockout and NCOA1-overexpressing mammary tumors, respectively, in several breast cancer mouse models. Knockout or knockdown of NCOA1 in breast cancer cell lines also markedly compromised their capability to induce angiogenesis in Matrigel plugs embedded subcutaneously in mice, while this compromised capability could be rescued by VEGFa treatment. At the molecular level, NCOA1 upregulates *VEGFa* expression in both mouse mammary tumors and cultured breast cancer cells, and it does so by associating with both c-Fos, which is recruited to the AP-1 site at bp −938 of the *VEGFa* promoter, and HIF1α, which is recruited to the HIF1α-binding element at bp −979 of the *VEGFa* promoter, to enhance *VEGFa* transcription. In 140 human breast tumors, high NCOA1 protein correlates with high MVD and patients with both high NCOA1 and high MVD showed significantly shorter survival time. In summary, this study revealed a novel mechanism that NCOA1 potentiates breast cancer angiogenesis through upregulating HIF1α and AP-1-mediated *VEGFa* expression, which reinforces the rational of targeting NCOA1 in controlling breast cancer progression and metastasis.

## INTRODUCTION

The nuclear receptor coactivator 1 (NCOA1 or SRC-1) belongs to the p160 SRC family that also includes NCOA2 (TIF2, GRIP1, SRC-2) and NCOA3 (SRC-3, AIB1, RAC3, ACTR) [1, 2]. These coactivators interact with nuclear receptors and certain other transcription factors (TFs) to recruit acetyltransferases such as CBP and p300 and methyltransferases such as CARM1 and PRMT1 to the enhancer for programing histone codes associated with active transcription [3–5]. These SRC coactivators not only play pivotal roles in development, growth, reproduction and metabolism, but also play crucial roles in cancer [2]. The oncogenic role of NCOA3 has been well established in various animal models and human cancers such as breast, prostate, colorectal and endometrial cancers [2, 6–8]. In breast cancer, NCOA3 is overexpressed and its overexpression is associated with HER2 activation, endocrine therapy resistance and poor disease-free survival (DFS) [2, 6, 9]. Knockout of Ncoa3 in mice significantly inhibits H-ras-, HER2- and chemical carcinogen-induced mammary tumorigenesis [10, 11]. Accordingly, overexpression of NCOA3 causes spontaneous development of mammary tumors [12]. At the molecular level, NCOA3 up-regulates the expression of multiple genes including cyclin D1, MMP-2, MMP-9 and macrophage migration inhibitory factor (MIF) [2, 13, 14]. Furthermore, SRC-3delta4, a splicing variant of NCOA3, can bridge EGFR to phosphorylate and activate FAK, which potentiates cancer cell survival, proliferation, migration and invasion [15]. NCOA1 is also overexpressed in 19–29% of human breast tumors and its overexpression positively correlates with HER2 expression, lymph node metastasis, disease recurrence and poor survival [16–18]. Although overexpression of NCOA1 in the mouse mammary gland is not oncogenic by itself and is also incapable of promoting oncogene-induced tumor growth, it significantly increased lung metastasis in Tg(MMTV-PyMT) (transgenic mouse mammary tumor virus-polyoma middle T) and Tg(MMTV-Neu) breast cancer mouse models [19]. Inversely, knockout of Ncoa1 remarkably inhibits lung metastasis without affecting primary mammary tumor growth in Tg(MMTV-PyMT) mice [20]. Mechanistically, NCOA1 has been shown to up-regulate Twist1, ITGA5, CSF-1, SDF1 and CXCR4 expression, which are partially responsible for promoting metastasis through potentiating breast tumor cell epithelial-mesenchymal transition (EMT), migration, invasion and macrophage recruitment [19, 21–23]. However, the specific role of NCOA1 in breast tumor angiogenesis, the hallmark of breast cancer progression to metastasis, remains to be defined.

Angiogenesis happens under both physiological conditions and pathological conditions [24]. Since tumor angiogenesis is required not only for providing oxygen and nutrients to support tumor growth but also for mediating tumor cell dissemination and metastasis to distant organs, it has been regarded as a fundamental step for a benign solid tumor to become a more malignant tumor [25]. Many angiogenic factors including PDGF, FGF, VEGF, TGFβ, Ang1, Ang2, VE-cadherin, CD31 and plasminogen activators have been demonstrated to promote endothelial proliferation and differentiation, recruit vascular smooth muscle cells, remodel extracellular matrix and stabilize vascular structures [26]. The VEGF family with five members including VEGFa, VEGFb, VEGFc, VEGFd and PGF are crucial angiogenic factors [27]. VEGFa is frequently overexpressed in various human solid tumors [28], which binds and activates its transmembrane tyrosine kinase receptors to promote endothelial proliferation, migration and invasion as well as vascular permeability [29, 30]. In addition, tumor cell-produced VEGFs can accelerate tumor cell progression and metastasis through stimulating tumor cell survival, migration and invasion, suppressing immune response and facilitating tumor cells homing to the bone marrow progenitors [31]. Several VEGF-targeted agents such as Bevacizumab have been applied to cancer therapy with clinical benefits when used alone or in combination with chemotherapy [28]. However, acquired resistance and induction of tumor invasiveness upon these treatments have emerged as major drawbacks of these applications [32]. Clearly, deep insights into understanding the mechanisms responsible for the transcriptional regulation of major angiogenic factors will help to yield more effective reagents for inhibiting angiogenesis.

Hypoxia in growing tumors stabilizes HIF1α to up-regulate VEGF expression, which in turn stimulates tumor angiogenesis [33]. However, the coactivators that mediate HIF1α transcriptional activity have not been fully characterized. In this study, we report that NCOA1 works with transcription factors HIF1α and AP-1 (c-Jun/c-Fos) to promote VEGFα expression in breast cancer cells and drive breast tumor angiogenesis in both mouse and human breast tumors. Our findings suggest that NCOA1-promoted breast cancer metastasis may be related to its role in angiogenesis and thus NCOA1 may serve as a new molecular target for inhibiting breast tumor angiogenesis and metastasis.

## RESULTS

### Ncoa1 expression positively correlates with microvascular density (MVD) in mouse mammary tumors

**Figure 1:**
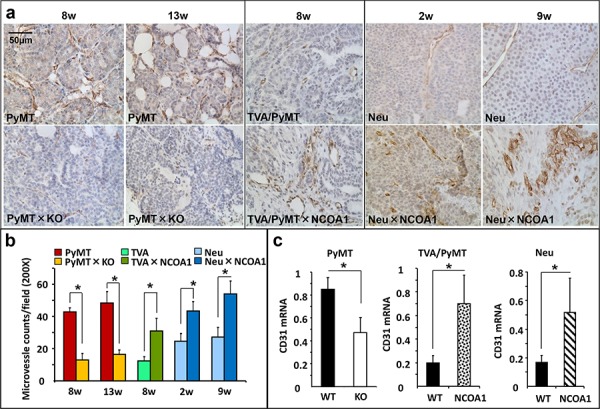
Microvascular density (MVD) in mouse mammary tumors with Ncoa1 knockout or overexpression **a.** Detection of CD31-positive endothelial cells by immunohistochemistry in mouse mammary tumor tissue sections prepared from Tg(MMTV-PyMT), Tg(MMTV-PyMT) × *Ncoa1*^−/−^, Tg(MMTV-TVA/RCAS-PyMT), Tg(MMTV-TVA/RCAS-PyMT) × Tg(MMTV-NCOA1), Tg(MMTV-Neu) and Tg(MMTV-Neu) × Tg(MMTV-NCOA1) mice as indicated. Tumors were isolated from mice after palpable tumors were detected for the time in weeks indicated. Scale bar: 50 μm. **b.** Semi-quantitative analysis of MVD. The total number of microvessels in 5 different viewing fields of 200× magnification under a microscope was counted for each tumor section. Sections from at least 10 tumors in each group were examined. Data are presented as Mean ± SD. *p < 0.05 by Student's *t* test. **c.** QPCR analysis of *CD31* mRNA in the mouse mammary tumors (*n* = 5) isolated from mice with the indicated genotypes. PyMT, Tg(MMTV-PyMT); TVA/PyMT, Tg(MMTV-TVA/RCAS-PyMT); Neu, Tg(MMTV-Neu); WT, wild type; NCOA1, Tg(MMTV-NCOA1).

To explore the relationship between Ncoa1 expression and mammary tumor angiogenesis, we examined MVD in mammary tumors developed in three previously established mouse models with normal *Ncoa1*, *Ncoa1* knockout or NCOA1 overexpression [19, 34–37] by immunostaining CD31, a molecular marker of endothelial cells. Semi-quantitative analysis revealed that MVD is reduced 70% and 60% in Ncoa1 knockout (Ncoa1^−/−^) mammary tumors versus Ncoa1 wild type (Ncoa1^+/+^) mammary tumors at week 8 and week 13 after the detection of palpable tumors in Tg(MMTV-PyMT) mice (Figure 1a and 1b). Consistently, MVD is remarkably increased in the mammary tumors with transgenic NCOA1 overexpression in Tg(MMTV-NCOA1) × Tg(MMTV-TVA/RCAS-PyMT) mice versus mammary tumors with normal Ncoa1 expression in Tg(MMTV-TVA/RCAS-PyMT) mice. In these mice, a subpopulation of the mammary epithelial cells with transgenic expression of TVA, a receptor for the RCAS avian virus, were specifically infected by the injected RCAS-PyMT avian virus and the infected cells were transformed into tumor cells by PyMT expression [19, 34]. Furthermore, MVD is also significantly increased in NCOA1-overexpressing mammary tumors in Tg(MMTV-NCOA1) × Tg(MMTV-Neu) mice versus mammary tumors with normal Ncoa1 expression in Tg(MMTV-neu) mice at both week 2 and week 9 after the detection of palpable tumors (Figure 1a and 1b). Moreover, quantitative RT-PCR (QPCR) analysis revealed that the relative expression levels of CD31 mRNA is significantly reduced in *Ncoa1*^−/−^ × Tg(MMTV-PyMT) mouse mammary tumors with Ncoa1 knockout, but significantly increased in Tg(MMTV-NCOA1) × Tg(MMTV-TVA/RCAS-PyMT) and Tg(MMTV-NCOA1) × Tg(MMTV-Neu) mouse mammary tumors with NCOA1 overexpression when compared with their respective control tumors described above (Figure 1c). On the other hand, CD31 expression showed no significant changes in the mammary tumors (*n* = 5) of *Ncoa3*^−/−^ × Tg(MMTV-PyMT) mice when compared with the mammary tumors (*n* = 5) of Tg(MMTV-PyMT) mice (data not shown). These observations suggest that the density of endothelial cells with CD31 expression positively correlates with the levels of Ncoa1 expression, but not Ncoa3 expression in the mouse mammary tumors. Together, these results demonstrate that the level of Ncoa1 expression positively correlates with MVD in all three different mouse breast cancer models.

### NCOA1 is required for breast cancer cell-stimulated angiogenesis *in vivo*

**Figure 2:**
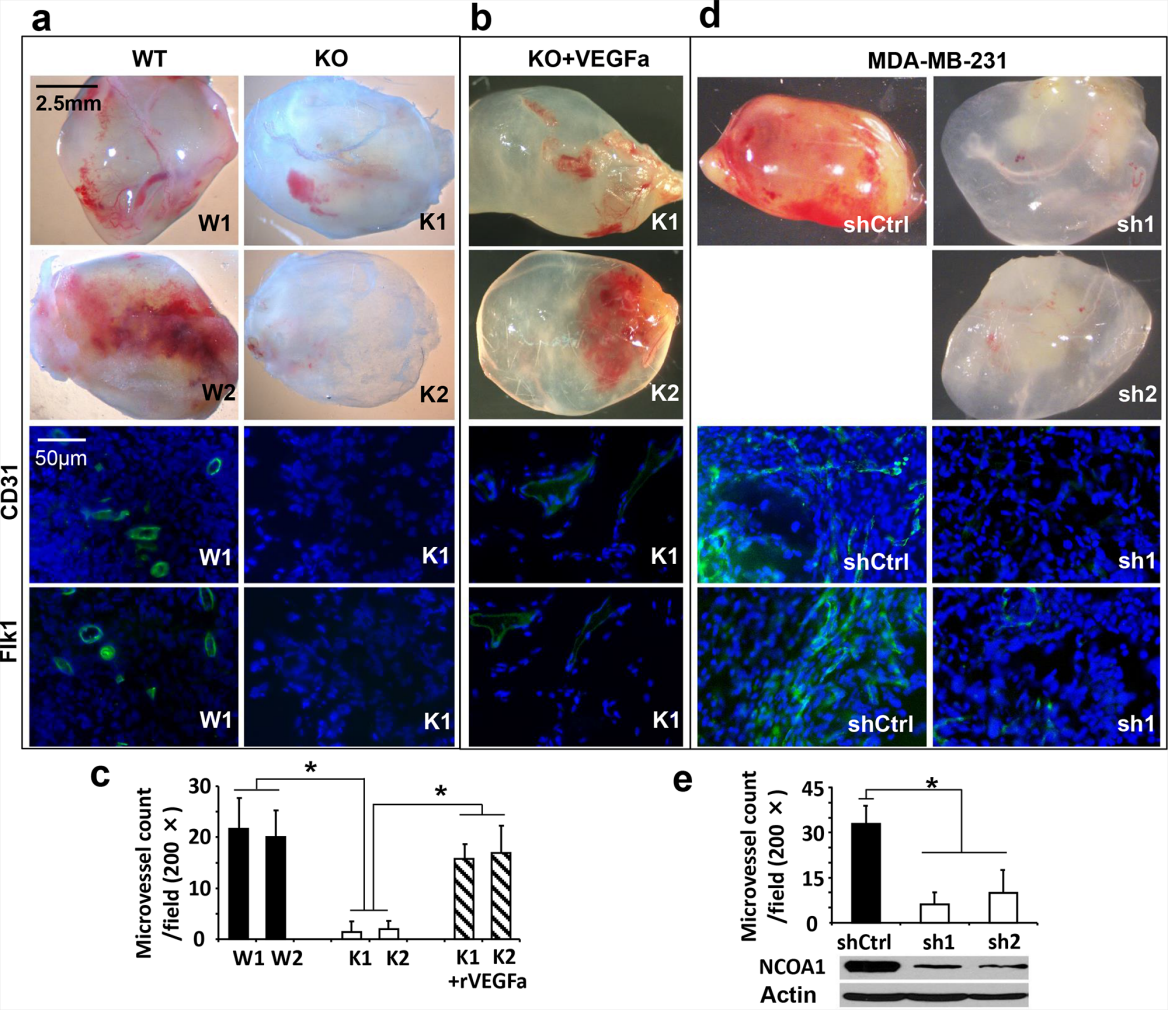
*In vivo* Matrigel angiogenesis induced by mouse and human breast tumor cells with Ncoa1 knockout and NCOA1 knockdown, respectively **a.** Representative images of Matrigel plugs with angiogenesis induced by W1, W2, K1 and K2 cells in SCID/beige mice (upper four panels) and representative images of CD31 and Flk1 immunofluorescent staining (green color) and DAPI staining (blue color) of Matrigel plug sections with angiogenesis induced by W1 and K1 cells (lower four panels). **b.** Representative images of Matrigel plugs with angiogenesis induced by K1 and K2 cells as well as 10 nM of recombinant VEGFa (rVEGFa) protein in Matrigel (upper two panels) and representative images of CD31 and Flk1 immunofluorescent staining and DAPI staining of Matrigel plug sections with angiogenesis induced by K1 cells plus rVEGFa (lower two panels). **c.** Semi-quantitative analysis of angiogenesis induced by W1, W2, K1 and K2 cells as well as K1 and K2 cells plus rVEGFa in Matrigel plugs in mice. A total of 12 Matrigel plugs with angiogenesis induced by the indicated cells with or without rVEGFa were analyzed. The number of microvessels per 200× viewing field was counted and 5 viewing fields were examined for each Matrigel plug. The data are presented as Mean ± SD. *p < 0.05 by Student's *t* test. **d.** Representative images of Matrigel plugs with angiogenesis induced by MDA-MB-231 cells expressing non-targeting shRNA (shCtrl) or NCOA1 mRNA-targeting shRNAs (sh1 and sh2) and representative images of CD31 and Flk1 immunofluorescent staining and DAPI staining of Matrigel plug sections with angiogenesis induced by MDA-MB-231 cells expressing shCtrl or sh1. **e.** Semi-quantitative analysis of angiogenesis induced by MDA-MB-231 cells expressing shCtrl, sh1 or sh2 in Matrigel plugs. Microvessels in 6 Matrigel plugs for each group were examined and counted as described above. Data are presented as Mean ± SD. *p < 0.05 by Student's *t* test. The knockdown efficiency of NCOA1 was analyzed by Western blot.

To determine whether Ncoa1 expressed in the mammary tumor cells supports angiogenesis, we performed in-gel-angiogenesis assay in mice to assess the angiogenesis-inducing capabilities of two Ncoa1 WT (W1 and W2) and two Ncoa1 knockout (K1 and K2) tumor cell lines, which were previously established from Tg(MMTV-PyMT) and Ncoa1^−/−^ × Tg(MMTV-PyMT) mouse mammary tumors [13, 21]. Subcutaneous injection of W1 or W2 cells into the clotted Matrigel plugs induced robust angiogenesis in the gel, while injection of same numbers of K1 or K2 cells induced very little angiogenesis under same conditions. In agreement with these observations, immunofluorescent staining for CD31 and Flk1, the two endothelial markers, identified many small blood vessels in the sections of Matrigel plugs injected with W1 or W2 cells, while the same staining only detected a few small blood vessels in the sections of Matrigel plugs injected with K1 or K2 cells (Figure 2a). The impaired angiogenesis in the gel plugs injected with K1 or K2 cells could be largely rescued by adding VEGFa into the Matrigel plugs, suggesting K1 and K2 cells do not produce enough VEGF for inducing angiogenesis (Figure 2b). Statistical analysis revealed significant decreases in MVDs induced by K1 or K2 cells versus that induced by W1 or W2 cells or by K1 or K2 cells supplemented with VEGFa (Figure 2c). Similarly, MDA-MB-231 human breast cancer cells expressing a non-targeting shRNA in the Matrigel plugs induced very active angiogenesis as indicated by the abundant red-colored blood vessels and CD31/Flk1-positive endothelial cells. However, MDA-MB-231 cells with stable knockdown of NCOA1 mRNA by expressing either of the two different shRNAs showed a very low capability to induce angiogenesis in the same Matrigel plugs (Figure 2d and 2e). These results demonstrate that NCOA1 expressed in both mouse and human breast cancer cells strongly promotes these cancer cell-induced angiogenesis *in vivo*.

### NCOA1 regulates VEGFa expression in breast cancer cells

**Figure 3:**
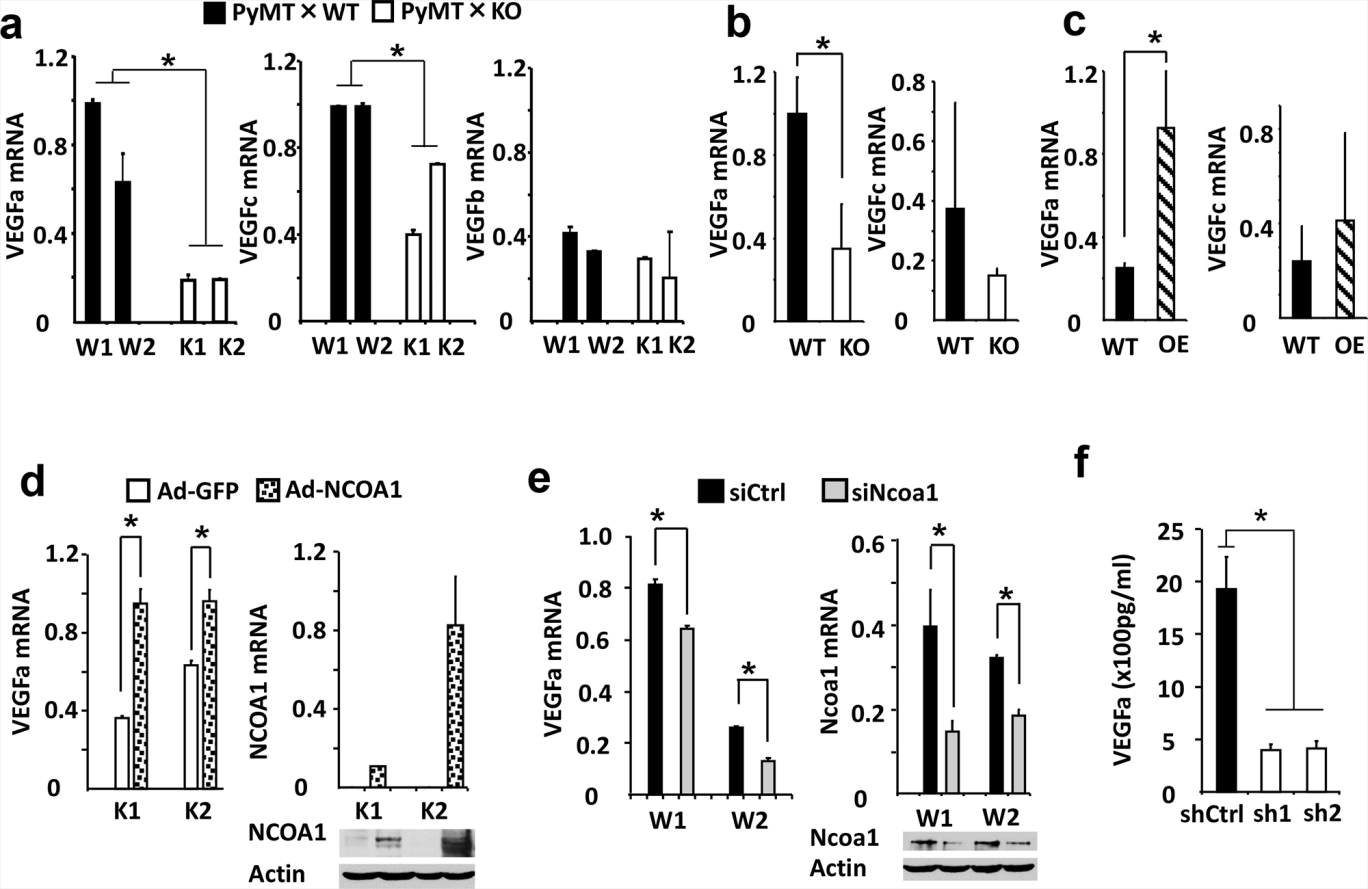
NCOA1 regulates VEGFa expression in breast tumor cells **a.** Relative expression levels of *VEGFa*, *VEGFc* and *VEGFb* mRNAs in W1, W2, K1 and K2 cells measured by QPCR. **b.** Relative expression levels of *VEGFa* and *VEGFc* mRNAs in Tg(MMTV-PyMT) (WT) and Tg(MMTV-PyMT) × *Ncoa1*^−/−^ (KO) mouse mammary tumors (*n* = 5) measured by QPCR. **c.** Relative expression levels of *VEGFa* and *VEGFc* mRNA levels in Tg(MMTV-TVA/RCAS-PyMT) (WT) and Tg(MMTV-TVA/RCAS-PyMT) × Tg(MMTV-NCOA1) (OE) mouse mammary tumors (*n* = 10) measured by QPCR. **d.** Relative expression levels of *VEGFa* mRNA in K1 and K2 cells with adenovirus-mediated GFP or NCOA1 expression (left panel). The expression levels of NCOA1 were analyzed by both QPCR and Western blotting (right panel). As expected, human *NCOA1* mRNA was not expressed in the mouse K1 and K2 cells. **e.** Relative expression levels of *VEGFa* and *Ncoa1* mRNAs in W1 and W2 cells transfected with siCtrl or Ncoa1 siRNAs as indicated. The measurement was carried out by QPCR. Ncoa1 knockdown efficiency was also analyzed by Western blotting. **f.** Secreted VEGFa concentrations in the conditioned media of MDA-MB-231 cells expressing non-targeting control shRNA (shCtrl) or NCOA1 mRNA-targeting shRNAs (sh1 and sh2). NCOA1 knockdown efficiency in these cells was shown in Figure 2e. The * in all panels indicates *p* < 0.05 by Student's *t* test.

To identify potential angiogenic factors regulated by Ncoa1, we measured the expression levels of many angiogenic factors in mouse mammary tumor cells that either have no functional Ncoa1 or have different levels of Ncoa1. We found that VEGFa mRNA expression is 5 and 3 fold lower in K1 and K2 Ncoa1 knockout mouse mammary tumor cells than that in W1 and W2 Ncoa1 WT mouse mammary tumor cells, respectively. VEGFc mRNA is also reduced 60% and 25% in K1 and K2 cells versus W1 and W2 cells, respectively. In contrast, VEGFb mRNA levels in K1 and K2 cells are comparable to that in W1 and W2 cells (Figure 3a). In addition, the expression levels of VEGFR1, VEGFR2, PDGFa, PDGFb, integrin α_v_β_3_, FGFR1 and FGFR2 in K1 and K2 cells are also similar to that in W1 and W2 cells (data not shown). In agreement with these results obtained from K1, K2, W1 and W2 cells in culture, VEGFa expression is also reduced more than 60% in Ncoa1 knockout mammary tumors in Ncoa1^−/−^ × Tg(MMTV-PyMT) mice versus Ncoa1 WT mammary tumors in Tg(MMTV-PyMT) mice. However, VEGFc expression levels showed no significant difference between these two types of mammary tumors (Figure 3b). Furthermore, the expression level of VEGFa mRNA, but not VEGFc mRNA, is increased more than 3 folds in NCOA1-overexpressing mammary tumors in Tg(MMTV-NCOA1) × Tg(MMTV-TVA/RCAS-PyMT) mice when compared with that in Ncoa1 WT mammary tumors in Tg(MMTV-TVA/RCAS-PyMT) mice (Figure 3c). Finally, we also measured VEGFa mRNA expressed in the mammary tumors with knockout of Ncoa3, another member of the SRC family. We found no significant difference in VEGFa mRNA expression levels between mammary tumors (*n* = 5) in Tg(MMTV-PyMT) × *Ncoa3*^−/−^ mice and mammary tumors (*n* = 5) in Tg(MMTV-PyMT) mice (data not shown). Together, the consensus of these results indicates that VEGFa expression levels positively correlate with Ncoa1 expression levels in multiple breast cancer mouse models.

To validate the regulatory relationship between NCOA1 and VEGFa in breast cancer cells, we further tested whether expression of NCOA1 in K1 and K2 Ncoa1 knockout cells could restore VEGFa expression and whether silencing NCOA1 expression in mouse and human breast cancer cells could down-regulate VEGFa expression. Indeed, adenovirus-mediated NCOA1 expression robustly increased VEGFa expression in both K1 and K2 cells (Figure 3d), while knockdown of Ncoa1 in W1 and W2 cells using a commercially available siRNA Smart Pool kit markedly reduced VEGFa expression (Figure 3e). Knockdown of NCOA1 using two different shRNAs in MDA-MB-231 human breast cancer cells also drastically reduced both VEGFa mRNA expression and VEGFa protein secreted into the culture medium (Supplementary Figure S1 and Figure 3f). These results indicate that NCOA1 either directly or indirectly regulates VEGFa expression in multiple types of breast cancer cells.

### NCOA1 is recruited to the proximal regions of the *VEGFa* promoter containing HIF1α and AP-1 binding elements

**Figure 4:**
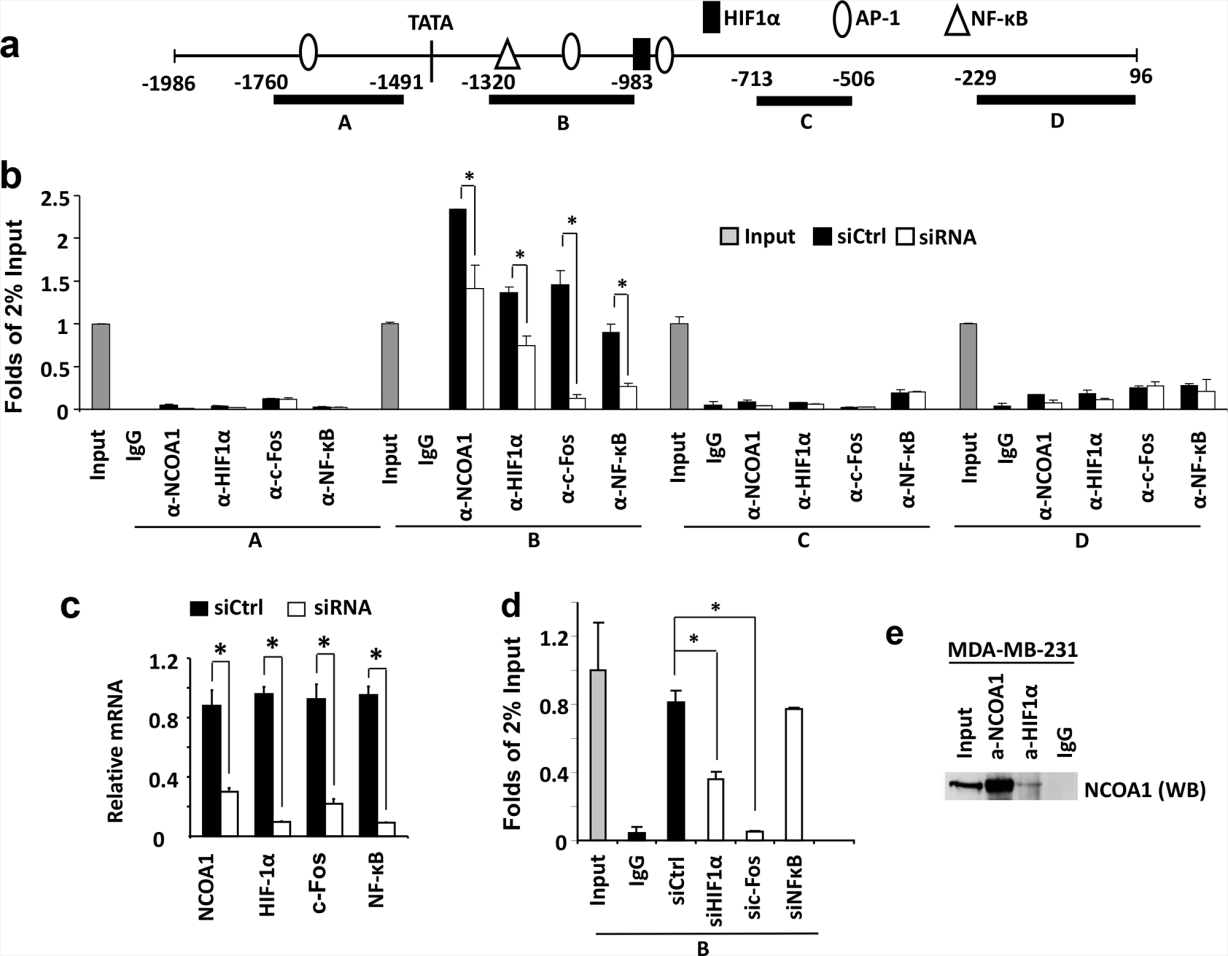
NCOA1 is recruited to the *VEGFa* promoter by HIF1α and c-Fos **a.** The *VEGFa* promoter region contains a TATA box and one HIF1a, three AP-1 and one NF-κB binding sites that are known to regulate *VEGFa* expression. Regions A–D were used for PCR amplification in ChIP assays. **b.** ChIP assays performed in MDA-MB-231 cells transfected with the non-targeting siRNA Smart Pool (siCtrl) or siRNA Smart Pools targeting *NCOA1, HIF1α, c-Fos or NF-κB* mRNAs as indicated. NCOA1, HIF1α, c-Fos and NF-κB antibodies were used for ChIP and normal IgG was used as negative control for ChIP. DNA obtained from ChIP was used as template for QPCR to measure the relative DNA amounts of regions A–D of the *VEGFa* promoter. The QPCR results for these regions were normalized to the QPCR results of 2% input DNA. **c.** The relative levels of *HIF1α, c-Fos and NF-κB* mRNAs in MDA-MB-231 cells transfected with non-targeting siRNA Smart Pool (siCtrl) or siRNAs targeting *NCOA1, HIF1α, c-Fos and NF-κB* mRNAs were measured by QPCR. **d.** ChIP assays for NCOA1-associated Region B of the *VEGFa* promoter in MDA-MB-231 cells with knockdown of HIF1α, c-Fos or NF-κB. Experiments in all panels were repeated at least three times. The * in all panels indicates *p* < 0.05 by Student's *t* test. **e.** Co-immunoprecipitation (Co-IP) assay for protein-protein interaction between NCOA1 and HIF1α. Cell lysate was prepared from MDA-MB-231 cells. Co-IP was performed with NCOA1 antibody, HIF1α antibody or non-immune IgG as negative control. The cell lysate for Co-IP (Input) and immunoprecipitated samples were analyzed by Western blotting (WB) using NCOA1 antibody.

It is known that several transcription factors including HIF1α, c-Fos and NF-κB are recruited to the proximal regions of the *VEGFa* gene promoter to activate *VEGFa* transcription (Figure 4a) [38–40]. Our chromatin immunoprecipitation (ChIP) assays confirmed that HIF1α, c-Fos and NF-κB are associated with a chromatin region (Region B in Figure 4a) that is downstream of the TATA box of the *VEGFa* promoter in MDA-MB-231 cells and this region contains one HIF1α binding element, two AP-1 elements and one NF-κB binding element. Furthermore, knockdown of HIF1α, c-Fos or NF-κB significantly reduced their recruitments to this chromatin region (Figure 4b), supporting the specific recruitments of these transcription factors to this chromatin region. In contrast, HIF1α, c-Fos and NF-κB are not associated with Region A with an AP-1 element and Regions C and D without any binding elements for these three transcription factors in the proximal regions of the *VEGFa* promoter (Figure 4a and 4b). Importantly, ChIP assays also revealed an efficient recruitment of NCOA1 to Region B of the *VEGFa* promoter in MDA-MB-231 cells, and this specific association was confirmed by its decreased recruitment to this region in MDA-MB-231 cells with NCOA1 knockdown (Figure 4b and 4c). No NCOA1 was found to be associated with the proximal Regions A, C and D of the *VEGFa* promoter (Figure 4b). These results demonstrate that NCOA1 is associated with the same chromatin region that also associated with transcription factors activating *VEGFa* expression.

To examine whether NCOA1 is recruited to chromatin Region B next to the *VEGFa* promoter by HIF1α, c-Fos and/or NF-κB, we performed ChIP assays using NCOA1 antibody in MDA-MB-231 cells with normal or silenced expression of HIF1α, c-Fos or NF-κB. Again, in MDA-MB-231 control cells transfected with a pool of non-targeting siRNAs, NCOA1 is efficiently recruited to the chromatin Region B (Figure 4c and 4d). When HIF1α or c-Fos expression is silenced by siRNA transfection, the recruitment of NCOA1 to Region B is markedly reduced. In contrast, knockdown of NF-κB expression does not affect NCOA1 recruitment to the chromatin Region B proximal to the *VEGFa* promoter (Figure 4c and 4d). It has been reported that SRC-1 (NCOA1) physically interacts with c-Jun and c-Fos to activate gene transcription [41]. Our co-immunoprecipitation assay also demonstrated that NCOA1 forms a protein complex with HIF1α in MDA-MB-231 breast cancer cells (Figure 4e). Together, these results demonstrate that NCOA1 is mainly recruited to a proximal region of the *VEGFa* promoter by associating with c-Jun, c-Fos and HIF1α.

### NCOA1 enhances HIF1α and AP-1 mediated activation of the VEGFa promoter

**Figure 5:**
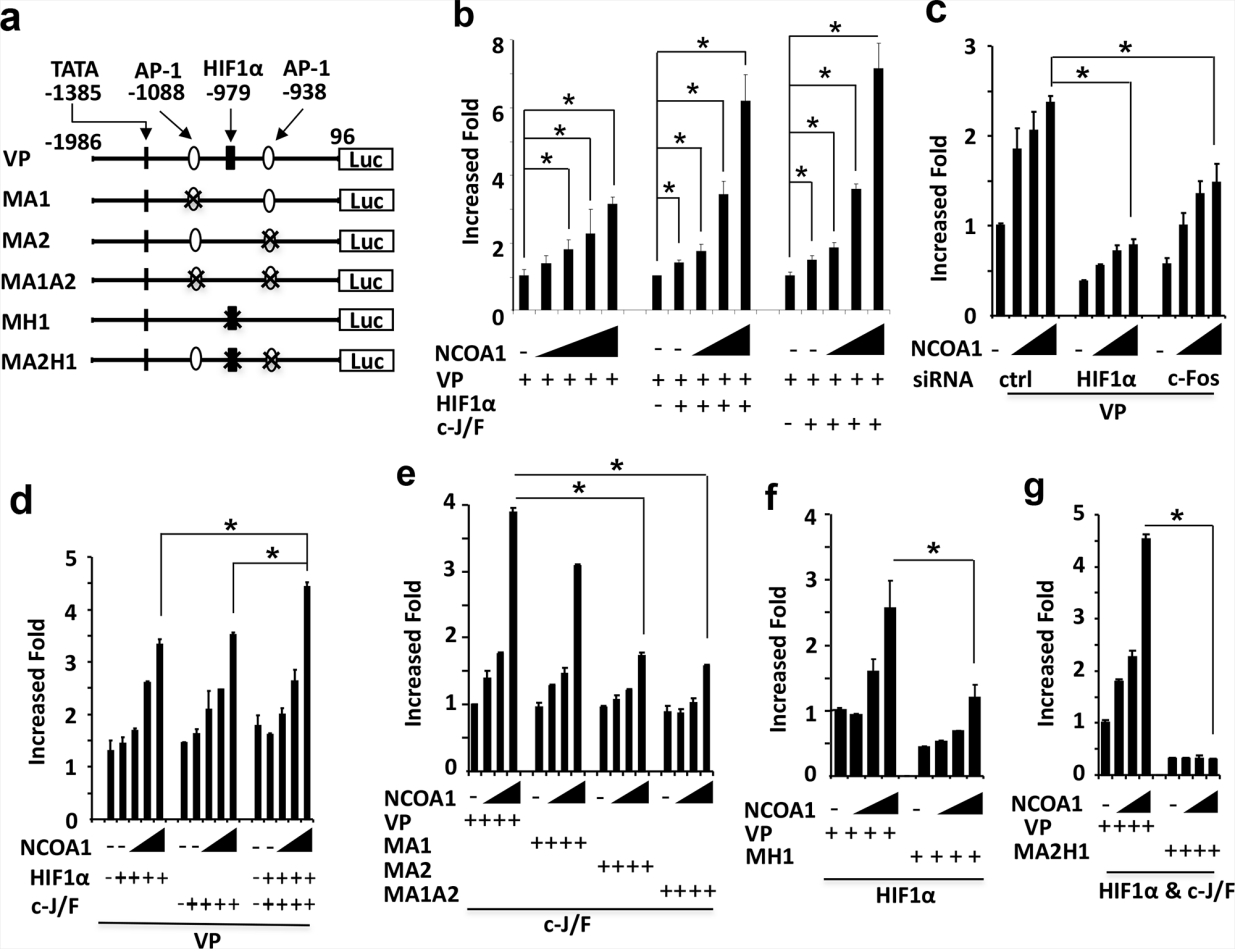
NCOA1 cooperates with HIF1α and AP-1 to activate the *VEGFa* promoter **a.** Wild type and mutant *VEGFa* promoter-reporter constructs. The locations are labeled by setting the transcriptional starting site as bp 1. TATA box and AP-1 and HIF1α binding sites are indicated. VP, wild type *VEGFa* promoter; MA1 or MA2, mutant *VEGFa* promoters with deletion of the first or second AP-1 site; MA1A2, mutant *VEGFa* promoter with deletion of both AP-1 sites; MH1, mutant *VEGFa* promoter with deletion of the HIF1α binding site; MA2H1, mutant *VEGFa* promoter with deletion of the HIF1α and the second AP-1 binding sites; Luc, luciferase. **b.** Enhancement of *VEGFa* promoter activity by NCOA1 alone or NCOA1 with HIF1α or c-Jun/c-Fos (c-J/F). HeLa cells in 24-well plates were co-transfected with 200 ng of VP-Luc plasmid and 0, 150, 300, 600, 900 or 1200 ng of NCOA1 expression plasmid (left panel) or 0, 0, 150, 300 and 600 ng of NCOA1 expression plasmid with 100 ng of HIF1α expression plasmid (middle panel) or c-J/F (50 ng each, right panel) expression plasmids as indicated. **c.** Knockdown of HIF1α or c-Fos reduced NCOA1-promoted activity of the *VEGFa* promoter. HeLa cells were transfected with 0, 150, 300 and 600 ng of NCOA1 expression plasmid and 200 ng of VP-Luc reporter plasmid. **d.** NCOA1 promotes HIF1α and c-J/F mediated activation of the *VEGFα* promoter. HeLa cells were co-transfected with VP-Luc reporter, NCOA1 and HIF1α, c-Jun/c-Fos or both HIF1α and c-Jun/c-Fos as described above for panel c. **e–f.** Deletion of the second AP-1 site or the HIF1α binding site reduced NCOA1/C-J/F or NCOA1/HIF1α-promoted activity of the *VEGFa* promoter. HeLa cells in 24-well plates were co-transfected with same amounts of NCOA1 plasmid as that in panel c, 100 ng of c-J/F plasmids or HIF1α plasmid, and 200 ng of VP-Luc, MA1-Luc, MA2-Luc or MA1A2-Luc reporter plasmid as indicated. **g.** Deletion of both the second AP-1 and the HIF1α binding sites diminishes NCOA1-enhanced HIF1α and C-J/F-mediated activation of the *VEGFa* promoter. HeLa cells were transfected with the indicated plasmids as described above for panel **f.** In all experiments, luciferase activity was assayed 48 hours after transfection and normalized to the total protein amount assayed for each sample. All experiments were repeated at least three times. The * in all panels indicates *p* < 0.05 by One-Way ANOVA test.

To determine whether NCOA1 could enhance the activity of the *VEGFa* promoter, we constructed a *VEGFa* promoter-luciferase (VP-Luc) reporter by using a 2 kb DNA fragment (bp −1986 to 96) known to contain sufficient *VEGFa* promoter activity [42] and DNA elements associated with HIF1α, c-Fos and NCOA1 (Figure 4, and Figure 5a). Expression of NCOA1 in HeLa cells increased the activity of VP-Luc reporter in a dose-dependent manner. Expression of either HIF1α or c-Fos with c-Jun in HeLa cells also slightly increased the activity of VP-Luc reporter. Importantly, co-expression of NCOA1 and HIF1α or NCOA1, c-Fos and c-Jun synergistically increased the activity of VP-Luc reporter (Figure 5b). Conversely, knockdown of HIF1α or c-Fos in HeLa cells significantly reduced NCOA1-promoted activity of VP-Luc reporter (Figure 5c). We also noticed that coexpression of NCOA1 with HIF1α, c-Fos and c-Jun in HeLa cells only slightly increased the activity of VP-Luc reporter when compared with co-expression of NCOA1 with HIF1α alone or with c-Fos and c-Jun (Figure 5d). This might be attributed to the limited availability of other factors required for further activating the reporter when NCOA1, HIF1α, c-Fos and c-Jun were all overexpressed in these cells. Together, these results strongly support the notion that NCOA1 serves as a coactivator for HIF1α and c-Fos to enhance the promoter activity of the *VEGFa* gene.

There are two AP-1 sites and one HIF1α-binding element in Region B of the *VEGFa* promoter (Figure 4a and Figure 5a). To examine which AP-1 site is required for NCOA1-enhanced and c-Jun/c-Fos-mediated activation of the *VEGFa* promoter, we constructed MA1-Luc reporter with the deletion of the first AP-1 site at bp −1088, MA2-Luc reporter with the deletion of the second AP-1 site at bp −938, and MA1A2-Luc reporter with the deletion of both sites. Co-expression of NCOA1, c-Jun and c-Fos in HeLa cells comparably activated VP-Luc and MA1-Luc reporters, while this co-expression only slightly activated MA2-Luc and MA1A2-Luc reporters in HeLa cells (Figure 5e). These results suggest that the second AP-1 site is required for NCOA1, c-Jun and c-Fos to activate the *VEGFa* promoter.

To examine whether the HIF1α-binding element in Region B of the *VEGFa* promoter is required for NCOA1-promoted and HIF1α-mediated activation of the *VEGFa* promoter, we constructed MH1-Luc reporter in which the HIF1α-binding element at bp −979 was deleted (Figure 5a). Co-expression of NCOA1 and HIF1α in HeLa cells robustly increased the activity of the VA-Luc wild type reporter, but only slightly increased the MH1-Luc mutant reporter (Figure 5f). These results demonstrate that the HIF1α-binding element in Region B is required for NCOA1 and HIF1α-induced activation of the VEGFa promoter.

Furthermore, deletion of both the HIF1α-binding element at bp −979 and the second AP-1 site at bp −938 in Region B completely abolished NCOA1, HIF1α and c-Jun/c-Fos promoted activation of the *VEGFa* promoter in HeLa cells (Figure 5a and 5g). These results indicate that NCOA1 works with both HIF1a and c-Jun/c-Fos to activate the *VEGFa* promoter.

### NCOA1 expression positively correlates with MVD in human breast tumors and poor patient survival

**Figure 6:**
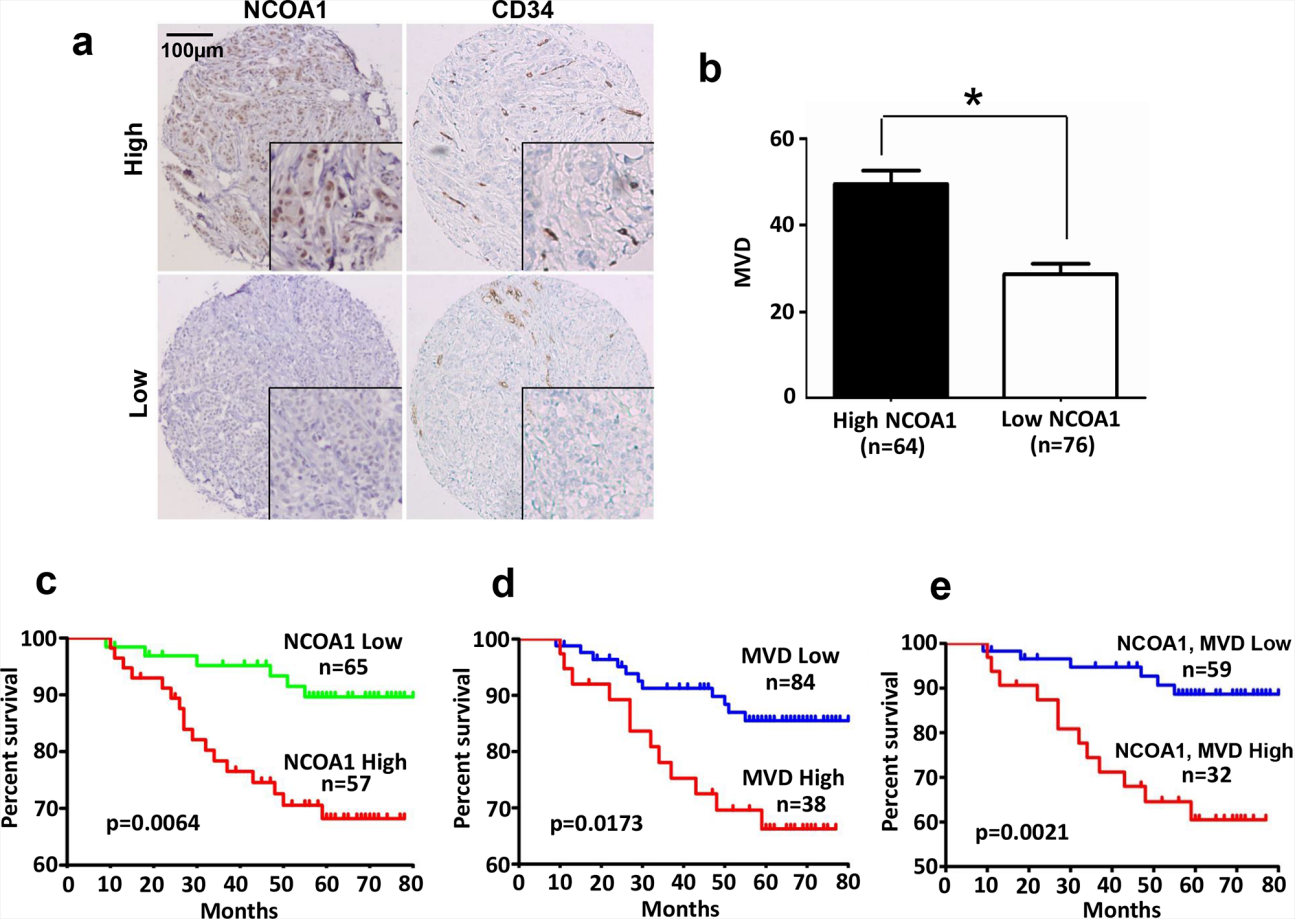
NCOA1 protein expression and its correlations with human breast tumor microvascular density (MVD) and patient survival **a.** Representative images of high and low immunoreactivities of NCOA1 and CD34 in breast tumor sections. Images were taken at the magnification of 200×. **b.** Semi-quantitative analysis of MVD in breast tumor tissues with high NCOA1 (immunoreactivity score ≥ 3) and low NCOA1 (immunoreactivity score < 3) protein expression. Data are presented as Mean ± SD microvessels per 200× viewing field. *P < 0.05 by Chi-square test. **c–d.** The Kaplan Meier survival curves of breast cancer patients with high versus low NCOA1 protein expression (panel c), with high MVD (>40) versus low MVD (≤40) (panel d), and with both high NCOA1 and high MVD versus both low NCOA1 and MVD (panel e). The indicated *p* values were calculated by Logrank test. n, number of patients.

Analysis of the RNA profiling data set obtained from a cohort of 270 human breast tumors, which was deposited in Oncomine Database by Bittner et al., revealed that the levels of *NCOA1* mRNA expression positively correlated with both expression levels of *VEGFa* mRNA (*r* = 0.23, *P* < 0.001) and CD31 mRNA (*r* = 0.40, *P* < 0.001) (Supplementary Figure S2). This is consistent with the role of NCOA1 in upregulating VEGFa to stimulate angiogenesis in human breast tumors. To validate this important role of NCOA1, we performed semi-quantitative immunohistochemistry (IHC) for NCOA1 in the tumor cells and CD34 in the vascular endothelial cells of small blood vessels in 140 human breast tumors. NCOA1 protein was mainly detected in the nuclei of breast cancer cells at different immunostaining intensities in different tumor sections. About 45% (64/140) and 55% (76/140) of breast tumors exhibited high (immunoreactivity score ≥ 3) and low (immunoreactivity score < 3) NCOA1 immunoreactivity. CD34 was detected in endothelial cells at similar immunostaining intensity, but different MVDs indicated by the CD34-positive cells were observed in different tumors (Figure 6a). We found that MVD in breast tumors expressing higher NCOA1 protein is significantly higher than that in breast tumors expressing lower NCOA1 protein (Figure 6b). These results confirmed that high NCOA1 expression is positively associated with high MVD in human breast tumors.

Kaplan-Meier survival curves were calculated for patient groups with high and low NCOA1 protein expression and MVD according to their long time-following up data. Patients with high NCOA1 protein or high MVD showed significantly worse overall survival rates versus patients with low NCOA1 protein or low MVD (Figure 6c and 6d). Patients with both high NCOA1 protein and high MVD showed the worst overall survival rate when compared with patients with both low NCOA1 protein and low MVD (Figure 6e). These results indicate that high NCOA1 expression concomitant with high MVD in breast tumors are associated with poor prognosis.

## DISCUSSION

In this study, we discovered that Ncoa1 protein levels are positively associated with the densities of small blood vessels in several models of mouse mammary tumors induced by PyMT or Neu (HER2) expression. Furthermore, we demonstrated that knockout of Ncoa1 in mouse mammary tumor cells or knockdown of NCOA1 in human breast cancer cells largely compromised their capabilities to induce angiogenesis *in vivo*. Moreover, we found that higher NCOA1 protein in human breast tumors is also positively associated with higher densities of small blood vessels. Patients with both high NCOA1 and high MVD exhibit the worst prognosis versus patients with low NCOA1, low MVD, and both low NCOA1 and low MVD. These observations indicate that NCOA1 plays an important role in breast cancer progression through promoting breast tumor angiogenesis. Tumor vascular density has been suggested as a clinical parameter for predicting recurrence and deciding treatment strategies [43]. In comparison with normal blood vessels, the tumor vessels are more tortuous and disordered, lack the clear hierarchy of arterioles, capillaries and venules, and have loose connections among endothelial cells, pericytes and basement membrane [44]. Since these features of tumor vessels facilitate the intravasation of tumor cells for metastasis, high vascular density (MVD) in tumors usually predicts worse outcomes in cancer patients [45]. Consistently, we have previously shown that NCOA1 overexpression in the mouse mammary tumor cells significantly promoted lung metastasis [19], while knockout of Ncoa1 in the mouse tumor cells drastically inhibited lung metastasis [20]. Similarly, breast tumors with high NCOA1 protein also showed poor prognosis (Figure 6c) [18, 46]. Therefore, NCOA1 overexpression-induced angiogenesis in breast tumors may directly contribute to breast cancer progression and metastasis.

Tumors induce angiogenesis by secreting various growth factors, and VEGFa is the most important one of such growth factors [47, 48]. In this study, we have identified *VEGFa* as a direct target gene of NCOA1 by multiple lines of evidence. First, we showed that the compromised ability of Ncoa1 null mammary tumor cells to induce *in vivo* angiogenesis can be rescued by VEGFa treatment, suggesting a reduced secretion of VEGFa from Ncoa1 null cells. Furthermore, we demonstrated that VEGFa expression in and secretion from breast tumor cells are positively associated with the manipulated NCOA1 expression levels, suggesting that NCOA1 tightly regulates *VEGFa* expression in these tumor cells. Moreover, NCOA1 is recruited to the *VEGFa* promoter by associating with HIF1α and c-Fos in breast cancer cells. Finally, we showed that NCOA1 potentiates the transcriptional activity of the *VEGFa* promoter by serving as a coactivator for both HIF1α and c-Fos/c-Jun that bind to the HIF1α binding site at bp −979 and the AP-1 site at bp −938 of the *VEGFa* promoter. Together, these findings indicate that the angiogenic function of NCOA1 in breast tumor is mediated, in part, by serving as a transcriptional coactivator for both HIF1α and AP-1 mediated *VEGFa* expression, since the crucial role of VEGFa in both physiological and pathological angiogenesis has been extensively studied and well documented [26, 49].

Although multiple transcription factors including HIF1α, AP-1, NF-κB, Sp1 and ERα have been reported to regulate VEGFa expression [38–40, 50, 51], our study is the first to show NCOA1, a transcriptional coactivator, can robustly and simultaneously coactivate two transcription factors, HIF1α and AP-1, to augment *VEGFa* expression and breast tumor angiogenesis. Our data showed that preventing either HIF1α or AP-1 from binding to the *VEGFa* promoter partially reduced NCOA1-promoted transcriptional activity of this promoter, while preventing both HIF1α and AP-1 from binding to the *VEGFa* promoter completely abolished NCOA1-promoted transcriptional activity of this promoter. This strongly supports that NCOA1 works with both HIF1α and AP-1 to upregulate *VEGFa* expression. On the other hand, co-expression of both HIF1α and AP-1 only slightly increased NCOA1-promoted transcriptional activity of the *VEGFa* promoter when compared with only expression of either HIF1α or AP-1, suggesting that other factors required for VEGFa transcription might become limited when HIF1α, AP-1 and NCOA1 are all overexpressed in the cell. Alternatively, considering there are only 23 base pairs of nucleotides interval between HIF1α and AP-1 binding sites in the promoter, the occupation of one site by either transcription factor might reduce the occupation of the other adjacent site by the other transcription factor. HIF1α in tumor cells is induced and activated under hypoxia condition, while AP-1 functions under both normoxia and hypoxia conditions. This explains why NCOA1 could enhance the transcriptional activity of the *VEGFa* promoter under both hypoxia and normoxia conditions and also implies a role of NCOA1 in upregulating *VEGFa* expression and tumor angiogenesis under both conditions.

NCOA1 works not only with both HIF1α and AP-1 to upregulate VEGFa expression as shown in this study but also works with different transcription factors to upregulate different target genes to promote breast cancer progression and metastasis as reported previously [19, 21, 22]. We have previously shown that NCOA1 interacts with PEA3 or AP-1 to upregulate the expression of Twist or integrin α5 and CSF-1. These target gene products further potentiate, either directly or indirectly, EMT, migration, invasion and/or metastasis of breast cancer cells [19, 21, 22]. These findings provide a possibility to use NCOA1 as a molecular target for inhibiting its multiple target gene-mediated pathways that drive breast cancer progression and metastasis. Especially, Ncoa1 knockout mice grow well and show a normal life span [35], supporting the notion that NCOA1 may be a preferential cancer target with tolerable adverse effect. Recently, gossypol and bufalin have been identified as specific small molecular inhibitors for both NCOA1 and NCOA3 and have been shown to inhibit breast cancer cell growth and/or metastasis in culture and/or mice [52, 53]. These translational studies suggest that targeting these overexpressed coactivators like NCOA1 and NCOA3 is indeed a feasible approach to control breast cancer growth and/or metastasis.

It is well established that tumor growth requires angiogenesis for supplying oxygen and nutrients [24]. However, we found that although NCOA1 promotes angiogenesis in breast tumors, overexpression or knockout of Ncoa1 in mice does not significantly affect mammary tumor growth [19, 20]. Although the underlying mechanisms are currently unknown, several possibilities may be speculated. First, we found that although Tg(MMTV-TVA/RCAS-PyMT) tumors grow much faster than Tg(MMTV-Neu) and Tg(MMTV-PyMT) tumors [19, 20], MVD in Tg(MMTV-TVA/RCAS-PyMT) tumors is actually much lower than MVD in the other two types of tumors (Figure 1b). These findings suggest that the growth of different types of tumors may have different requirements on their MVDs. It is possible that breast tumor cells with different levels of Ncoa1 expression also have different requirements on MVD for growth. Furthermore, the changes of other Ncoa1 functions independent of its angiogenic function caused by the changed Ncoa1 expression levels may counter-regulate the growth effects of angiogenesis in breast tumors. For example, knockout of Ncoa1 influences fat oxidation and energy metabolism [54, 55], which may complicate the correlation relationship between Ncoa1-promoted tumor angiogenesis and Ncoa1-influenced tumor growth.

## MATERIALS AND METHODS

### Breast cancer mouse models and cell culture

The original Tg(MMTV-PyMT), Tg(MMTV-TVA/RCAS-PyMT), Tg(MMTV-Neu), Tg(MMTV-NCOA1) and *Ncoa1*^−/−^ mouse lines were described previously [19, 34–37]. The Tg(MMTV-PyMT), Tg(MMTV-PyMT) × *Ncoa1*^−/−^, Tg(MMTV-TVA/RCAS-PyMT), Tg(MMTV-TVA/RCAS-PyMT) × Tg(MMTV-NCOA1), Tg(MMTV-Neu) and Tg(MMTV-Neu) × Tg(MMTV-NCOA1) mice used in this study were produced as described previously [19, 20]. The MMTV-PyMT mouse mammary tumor cell lines with Ncoa1 expression including W1 and W2 cell lines and with Ncoa1 knockout including K1 and K2 cell lines were developed from mammary tumors in Tg(MMTV-PyMT) and Tg(MMTV-PyMT) × *Ncoa1*^−/−^ mice and cultured as described previously [21]. MDA-MB-231 human breast cancer cells and HeLa cells were cultured in DMEM containing 10% FBS.

### Mouse mammary tumor collection, processing and immunohistochemistry

Mammary tumors were isolated from Tg(MMTV-PyMT) and Tg(MMTV-PyMT) × *Ncoa1*^−/−^ mice at week 8 and week 13 after their palpable tumors were detected, from Tg(MMTV-TVA/RCAS-PyMT) and Tg(MMTV-TVA/RCAS-PyMT) × Tg(MMTV-NCOA1) mice at week 13 after their palpable tumors were detected, and from Tg(MMTV-Neu) and Tg(MMTV-Neu) × Tg(MMTV-NCOA1) mice at week 2 and week 9 after their palpable tumors were detected. The mammary tumor tissues were fixed in 4% paraformaldehyde (PFA) in phosphate-buffered saline (PBS), dehydrated in ethanol solution series and embedded in paraffin blocks. Tissue sections were prepared and immunohistochemistry was performed with FLK and CD31 antibodies (ab 28364, Abcam, 1:200 dilution used) as described [13, 21].

### Knockdown and adenovirus-mediated expression of NCOA1

Lentiviral particles for expressing non-targeting control shRNA (shCtrl) and NCOA1 mRNA-targeting shRNAs including shNCOA1-1 (sh1) and shNCOA1-2 (sh2) were obtained from the Cell-Based Assay Screening Core at Baylor College of Medicine and used to infect MDA-MB-231 cells as described previously [22]. The infected cells were selected in the medium containing 1 μg/ml puromycin for 14 days to establish stable NCOA1-knockdown cells for experiments. To knock down mouse Ncoa1, the W1 and W2 cells were transiently transfected with siRNA Smart Pool that targets mouse Ncoa1 mRNA (Dharmacon, Inc. Lafayette, CO). The same cells transfected with a non-targeting siRNA pool were used as controls. Adenoviruses containing a GFP or a NCOA1 expression cassette were used to infect the K1 and K2 cells for expressing GFP as a control or NCOA1 as described previously [13, 56].

### Reporter construction and luciferase assay

The 5′ regulatory DNA fragment from bp −1987 to 96 of the human *VEGFa* gene was amplified by high-fidelity PCR using specific primers (Supplementary Table S1) and subcloned into the pGL3 basic vector with a luciferase reporter (Promega, Inc. Madison, WI). Mutant promoter-reporter plasmids with deleted HIF1α-binding site at bp −979 and/or AP-1-binding sites at bp −1088 and bp −938 were constructed by using the similar approaches as described previously [19]. HeLa cells were cultured in 6-well plates and transfected with 200 ng/well of the promoter-reporter plasmid, 100 ng/well of the HIF1α, NF-κB, or c-Jun and c-Fos expression plasmids and different amounts of NCOA1 expression plasmid as described previously [19]. Luciferase activity was also assayed as described previously [19]. In HIF1α-transfected cells, CoCl_2_ was added to 100 μM in the medium to create a hypoxia condition as described previously [57].

### *In vivo* matrigel angiogenesis assay

Experiment was performed as described previously [58]. Briefly, 150 μl of Matrigel (BD Biosciences, San Jose, CA) was subcutaneously injected to the back of 5–6-week-old female SCID/beige mice to form plugs. Then, 1 × 10^5^ of mouse mammary tumor or human breast cancer cells were injected into the central region of the Matrigel plugs. After the injected mice were maintained for 8 days, the Matrigel plugs were isolated for imaging. The imaged Matrigel plugs were fixed in 2% PFA, washed with PBS, and frozen in OCT at −80°C. Cryosections were prepared from the frozen samples and these sections were processed for immunofluorescent staining using CD31 and Flk1 antibodies.

### ELISA

The concentration of VEGFa protein was measured using human VEGFa ELISA kit (Invitrogen, Grand Island, NY) by following the manufacturer's instructions. Twenty μl of medium (DMEM containing 0.5% FBS) conditioned by MDA-MB-231 cells expressing non-targeting shRNA or NCOA1 shRNA for 48 hours was used for each measurement.

### ChIP assay

ChIP assays were performed as described previously [21, 22]. Briefly, MDA-MB-231 cells with and without knockdown of NCOA1, HIF1α, c-Fos or NF-κB were fixed in 1% formaldehyde for 10 minutes. DNA-protein complexes extracted from these fixed cells were subjected to immunoprecipitation with NCOA1, HIF1α, c-Fos or NF-κB antibodies. Immunoprecipitated DNA was eluted and subjected to QPCR analysis using specific primer pairs (Supplementary Table S1) that amplify different promoter regions indicated in Figure 4. In negative control ChIP assays, equal amount of normal IgG was used to replace specific antibodies. Each experiment was repeated at least three times.

### Microvascular density determination

Microvascular density was determined as described previously [59]. In brief, blood vessels were visualized by CD31 or CD34 immunohistochemistry or immunofluorescence in the tissue sections prepared from mouse and human breast tumors and Matrigel plugs with angiogenesis. Tumor areas within the “hot spots” regions, which contain greatest vessel density, were imaged under a microscope at 200× magnification. Tumor areas associated with ulceration or granulation were excluded from consideration as the “hot spot”. Any stained endothelial cell was counted to represent a single vessel if it was clearly separated from adjacent microvessels and other connective tissue elements. Two independent scientists performed the vessel density evaluation and the average was regarded as the final microvessel count.

### Breast cancer patients and tissue microarray

A total of 140 human breast tumor specimens were collected from primary surgeries before any endocrine therapy was given during 2006–2008 at Southwest Hospital of Third Military Medical University in China. 122 patients were followed up by clinic interviews for 9–80 months. Patient demographic and pathohistologic data including age, tumor size and grade, lymph node metastasis, recurrence and survival time, estrogen and progesterone receptor expression, as well as HER2 expression were retrieved from clinical records. Tumor specimens embedded in the paraffin blocks were used for constructing tissue microarrays as described [13]. Immunohistochemistry using NCOA1 and CD34 antibodies was performed as described previously [13]. The immunostaining intensity for NCOA1 was independently scored by two pathologists according to the Allred scoring system [60] and the average score for each sample was regarded as the final score. MVD in tumors visualized by CD34 immunostaining was determined as described above. Pearson Chi-square test was used for categorical variables to compare two proportions. Kaplan Meier estimates of survival functions were computed and Logrank test was applied to compare the difference of survival. *P* < 0.05 was considered to be statistically significant. This study was approved by the Ethics Committee of Southwest Hospital of Third Military Medical University, and written informed consents were obtained from all patients prior to treatment.

### Other methods

QPCR, Western blotting and Co-immunoprecipitation were performed as described previously [21, 22]. Primer and universal probe sequences for QPCR were provided in Supplementary Table S1.

## SUPPLEMENTARY MATERIAL FIGURES AND TABLES


