# Supplementary material for: NCOA1 promotes angiogenesis in breast tumors by simultaneously enhancing both HIF1α- and AP-1-mediated VEGFa transcription
Source: Oncotarget. 2015 Jun 20;6(27):23890–904. doi: 10.18632/oncotarget.4341 (PMC4695159; doi:10.18632/oncotarget.4341)
Supplement: Supplementary file 1 [file oncotarget-06-23890-s001.pdf]

SUPPLEMENTARY FIGURES AND TABLES

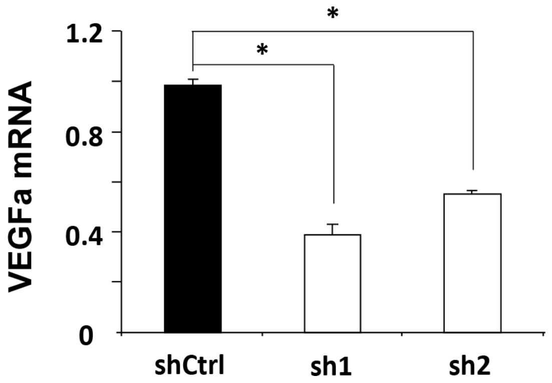

Supplementary Figure S1: NCOA1 was knocked down in MDA-MB-231 cells using shRNA targeting two different region (sh1 and sh2) and VEGFa mRNA levels were examined by qPCR. Scramble knockdown (shCtrl) served as the control.

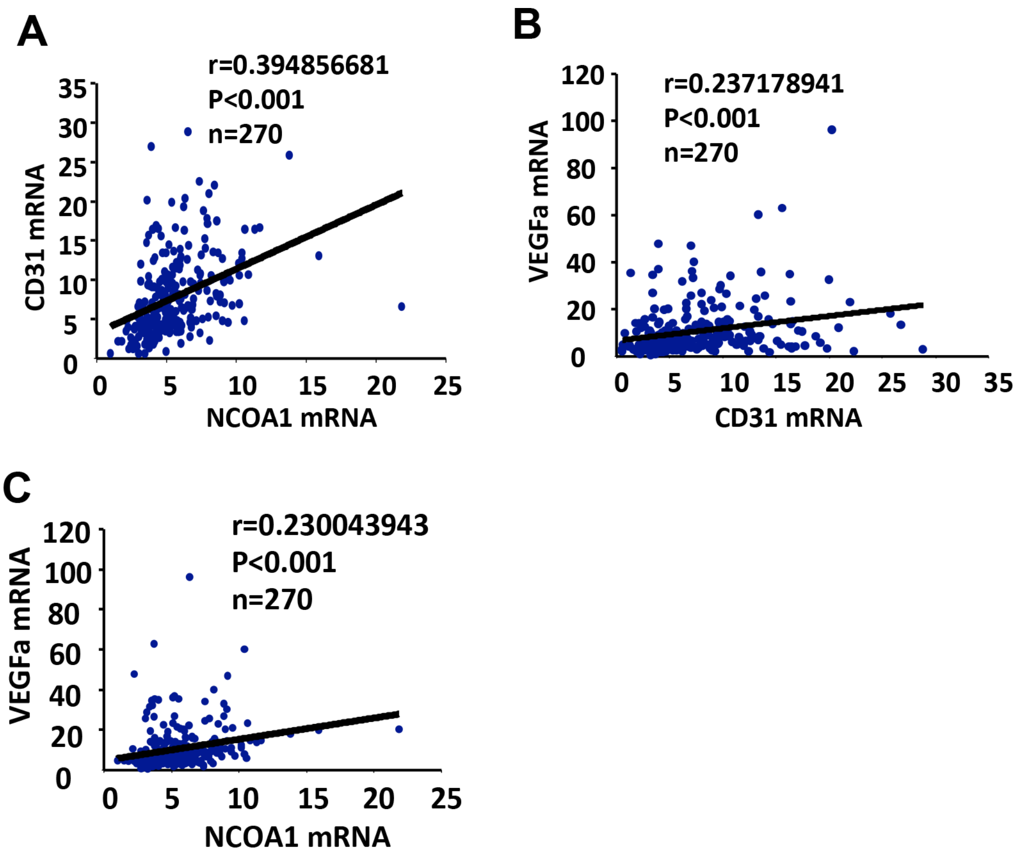

Supplementary Figure S2: The expression levels of *NCOA1*, *CD31* and *VEGFa* mRNAs are positively associated in a cohort of human breast tumors (*n* = 270). The whole genome mRNA expression profile was determined by microarray and deposited in the Oncomine Database (https://www.oncomine.com/resource/main.html) by Dr. Bittner M at International Genomics Consortium in Arizona. The correlation relationships in expression levels between *NCOA1* and *CD31* mRNAs (panel a), between *CD31* and *VEGFa* mRNAs (panel b), and between *NCOA1* and *VEGFa* mRNAs (panel c) were analyzed using Pearson Correlation Coefficient (*r* value) and statistical significance (*p* value).

Supplementary Table S1: PCR Primers and TaqMan QPCR Probes

Amplicons	Forward primers	Reverse primers	TaqMan probes
QPCR:			
mNcoa1[(1)]	tatctctccagcccatggtgt	caaagttcccttggttgttgc	tcatccacgttgccaccatcca
mCD31	gctggtgctctatgcaagc	atggatgctgttgatggtga	universal probe #64[(2)]
mVEGFa	gcagcttgagttaaacgaacg	ggttcccgaaaccctgag	universal probe #4
mVEGFb	cccagccaccagaagaaa	acattgcccatgagttccat	universal probe # 10
mVEGFc	aacacacagaagtgcttccttaaa	tcgcacacggtcttctgta	universal probe #31
mβ-actin	ctaaggccaaccgtgaaaag	accagaggcatacagggaca	universal probe #64
hNCOA1[(3)]	tgcagcagaatgtcttccag	aaagttggcctcaccttgg	universal probe #15
hVEGFa	cgcaagaaatcccggtataa	aaatgctttctccgctctga	universal probe #1
hHIF1α	tttttcaagcagtaggaattgga	gtgatgtagtagctgcatgatcg	universal probe #66
hc-Fos	actaccactcacccgcagac	ccaggtccgtgcagaagt	universal probe #67
hNF-κB	ctggcagctcttctcaaagc	tccaggtcatagagaggctca	universal probe # 22
hβ-actin	ccaaccgcgagaagatga	ccagaggcgtacagggatag	universal probe #64
PCR for ChIP assays:			
Region A (268 bps)[(4)]	ggacaccataccgatggaac	gaacaaagttggggctctga	
Region B (339 bps)	aagctgtgagcctggagaag	cactgtggagtctggcaaaa	
Region C (207 bps)	gaagcaactccagtcccaaa	cacacgtcctcactctcgaa	
Region D (325 bps)	ttttcaggctgtgaaccttg	ctgaccggtccacctaacc	
PCR for human VEGFa promoter:			
VP (bp -1986/96)	gttaggtaccatcccgttcatcagcctaga	gttaccatggcggctgaccggtccacctaacc	
MA1	ttagtgctggcgggtaggcacgcaggccct	tggaggccagggcctgcgtcctacccgcca	
MA2	aggtcctcttccctcccagtcggaaccaca	cgggaagctgtgtggttccgactgggaggga	
MH1	Ggttttgccagactccacagaacaggtcctc	ctgggagggaagaggacctgttctgtggagtc	

[(1)]mNcoa1, mouse Ncoa1;
[(2)]The universal probe for each mRNA was matched by searching the Universal ProbeLibrary (Roche Biotechnology) using the web-based ProbeFinder softwae (http://www.universalprobelibrary.com);
[(3)]hNCOA1, human NCOA1;
[(4)]bps, base pairs.
